# Evaluation of knowledge of nurses in intensive care, semi-intensive care and ready for a private hospital of St Paul on sepsis

**DOI:** 10.1186/cc10159

**Published:** 2011-06-22

**Authors:** CDC Gambin, DFM Junior, SCL Shiramizo, ACMA Gonçalves, E Silva

**Affiliations:** 1 Hospital Israelita Albert Einstein, Morumbi, São Paulo - SP, Brazil

## Background

Sepsis is an inflammatory response secondary to an infectious process with presumed or known [1] focus that can lead to involvement of multiple organs and death. The incidence of severe sepsis and septic shock among patients admitted to intensive care units (ICUs) in Brazil was 36 and 30 per 1,000 patient-days, respectively [2]. ICUs in other countries reported an incidence of severe sepsis of 21 cases per 100 admissions in Paris [3] and 16 cases per 100 admissions in the United States [4].

## Method

Field research, descriptive and exploratory, transversal, prospective, level I, with a quantitative approach. We approached nurses working in intensive care, emergency care and semi-intensive work during the day or night. The study was conducted in a large private hospital in São Paulo. A semi-structured questionnaire was developed with multiple-choice questions containing personal data and information on knowledge about sepsis.

## Results

We found 82 respondents with 33 nurses from the ICU, 30 from semi-intensive units (USI) and 19 of the health care unit (APU); there is a predominance of females and training time, being an average of 80%. Over 60% of respondents were postgraduates. The APU was found to have the greatest number of correct classifications of sepsis, more than 50% of respondents; the ICU was in second place, with an average of 40% hits; and the USI averaged 30% correct.

## Conclusion

Of the nurses responding to the questionnaire, 66 (80%) are female and 74 (90%) have worked for more than 1 year and are trained well, and 22 (26%) hold a postgraduate program graduation. According to the results, we can observe that the better performance was seen in the emergency care units and intensive care. This does not exclude such units from a proposal for continuing education, since the primary concern relates to the retention of clinical symptoms.
